# Curcumin induces therapeutic angiogenesis in a diabetic mouse hindlimb ischemia model via modulating the function of endothelial progenitor cells

**DOI:** 10.1186/s13287-017-0636-9

**Published:** 2017-08-03

**Authors:** Jinzhi You, Jiacheng Sun, Teng Ma, Ziying Yang, Xu Wang, Zhiwei Zhang, Jingjing Li, Longgang Wang, Masaaki Ii, Junjie Yang, Zhenya Shen

**Affiliations:** 1 Department of Cardiovascular Surgery of the First Affiliated Hospital & Institute for Cardiovascular Science, Soochow University, Suzhou, China; 2 Division of Research Animal Laboratory and Translational Medicine, Osaka Medical College, Osaka, Japan

**Keywords:** Curcumin, Diabetes mellitus, Endothelial progenitor cells, Hindlimb ischemia, Neovascularization

## Abstract

**Background:**

Neovascularization is impaired in diabetes mellitus, which leads to the development of peripheral arterial disease and is mainly attributed to the dysfunction of endothelial progenitor cells (EPCs). Previous studies proved the promotional effect of curcumin on neovascularization in wound healing of diabetes. Thus, we hypothesize that curcumin could promote neovascularization at sites of hindlimb ischemia in diabetes and might take effect via modulating the function of EPCs.

**Methods:**

Streptozotocin-induced type 1 diabetic mice and nondiabetic mice both received unilateral hindlimb ischemic surgery. Curcumin was then administrated to the mice by lavage for 14 days consecutively. Laser Doppler perfusion imaging was conducted to demonstrate the blood flow reperfusion. Capillary density was measured in the ischemic gastrocnemius muscle. In addition, angiogenesis, migration, proliferation abilities, and senescence were determined in EPCs isolated from diabetic and nondiabetic mice. Quantitative PCR was then used to determine the mRNA expression of vascular endothelial growth factor (VEGF) and angiopoetin-1 (Ang-1) in EPCs.

**Results:**

Curcumin application to type 1 diabetic mice significantly improved blood reperfusion and increased the capillary density in ischemic hindlimbs. The in-vitro study also revealed that the angiogenesis, migration, and proliferation abilities of EPCs and the number of senescent EPCs were reversed by curcumin application. Quantitative PCR confirmed the overexpression of VEGF-A and Ang-1 in EPCs after curcumin treatment.

**Conclusion:**

Curcumin could enhance neovascularization via promoting the function of EPCs in a diabetic mouse hindlimb ischemia model.

**Electronic supplementary material:**

The online version of this article (doi:10.1186/s13287-017-0636-9) contains supplementary material, which is available to authorized users.

## Background

Diabetic mellitus has a high risk of developing peripheral arterial disease (PAD) and critical limb ischemia [1], which is mainly attributed to hyperglycemia-induced impaired neovascularization. Neovascularization, including angiogenesis and vasculogenesis, plays a crucial role in the delivery of oxygen, nutrients, and other mediators at the injury and ischemic sites. Angiogenesis is a progress of stimulation, promotion, and stabilization of new blood vessels that involves major angiogenic factors such as vascular endothelial growth factor (VEGF) and angiopoetin-1 (Ang-1), but their levels decrease in diabetes [2, 3]. Vasculogenesis involves participation of endothelial progenitor cells (EPCs), a cell type that is capable of differentiating into functional endothelial cells and replacing the injured endothelial cells [4, 5]. During vasculogenesis, EPCs mobilize from bone marrow (BM), home to the ischemic site, differentiate into endothelial cells, and participate in endothelial repair [6, 7]. However, the number and function of EPCs are reduced in diabetes-associated peripheral vascular disease [8], which is a major cause of impaired vascular regeneration/maintenance under hyperglycemia conditions [9].

There are limited medical therapies to improve angiogenesis in diabetes associated with PAD [10]. Recently, Kant et al. [11] indicated that curcumin had a proangiogenic effect on wound healing in type 1 diabetes. Curcumin (C_2_H_20_O_6_) or diferuloylmethane is a bright-yellow chemical isolated from the roots of *Curcuma Longa* (turmeric), and has been widely used and studied over the past 60 years in the treatment of numerous diseases [12]. In addition to the proangiogenic effect, evidence also showed that curcumin had antiangiogenic effects on pituitary adenomas and hepatic cancer [13, 14]. Therefore, curcumin exhibited contradictory results in different settings of diseases.

Considering the limited studies of curcumin in diabetic ischemia, the present study was conducted to investigate the potential of curcumin on neovascularization in diabetic ischemia as well as the possible mechanism.

## Methods

### Animals

Healthy male C57/BL6 mice were housed in standard polycarbonate cages in the Animal Facility of Soochow University. Animals were maintained on a 12-hour light/dark cycle as well as provided with free access to feed and water. All animals received humane care in accordance with the Guidelines for the Care and Use of Research Animals established by Soochow University.

### Type 1 diabetes and hindlimb ischemia induction

Type 1 diabetes was induced by intraperitoneal injections of streptozotocin (Sangon Biotech, Shanghai, China). Male C57/B6 mice (4–6 weeks of age) received an injection of citrate buffer (4.92 mol/ml sodium citrate, pH 4.2–4.5) or streptozotocin (50 mg/kg) dissolved in sterile citrate buffer for 5 days consecutively. The blood glucose level was measured 7, 14, and 21 days after the injection. Mice with a blood glucose level > 12.0 mmol/L were considered diabetic and selected for experiments.

Three weeks after diabetes induction, all nondiabetic and diabetic mice received unilateral hindlimb artery devascularization. Mice aged 6–8 weeks were anesthetized with 160 mg/kg pentobarbital by intraperitoneal injection. During the operation, the superficial and deep femoral vessel, the common femoral vessel and its abdominal branches were ligated and excised to generate hindlimb ischemia as described previously [15]. The right femoral artery was exposed but not dissected to serve as the nonischemic control.

### Groups and curcumin treatment

Animals were divided equally into four groups. Twenty mice were used in this study, namely five mice for each group. In the nondiabetes group, mice with euglycemia were treated with 300 μl of sterile saline by lavage once a day for 14 days. In the diabetes with saline treatment group, sterile saline (300 μl) was applied to diabetic mice by lavage once a day for 14 days. In the diabetes with olive oil treatment group, totally 300 μl of extra virgin olive oil was administrated by lavage to each mouse once a day for 14 consecutive days. In the diabetes with curcumin treatment group, 1000 mg/kg curcumin (Sigma-Aldrich, St. Louis, MO, USA) in 300 μl of olive oil was applied to mice once a day for 14 days.

### Laser Doppler perfusion imaging

Laser Doppler perfusion imaging (LDPI) (Perimed Instruments AB, Stockholm, Sweden) was conducted on mice immediately, 7 days, and 14 days after ischemic surgery. LDPI was used to measure the blood flow recovery ratio in ischemic hindlimbs:

$$ \mathrm{Blood}\;\mathrm{flow}\;\mathrm{recovery}\;\mathrm{ratio}=\frac{\mathrm{Ischemic}\;\mathrm{limb}\;\mathrm{perfusion}\;\left(\mathrm{left}\;\mathrm{hindlimb}\right)}{\mathrm{Nonischemic}\;\mathrm{limb}\;\mathrm{perfusion}\;\left(\mathrm{right}\;\mathrm{hindlimb}\right)}\times 100\% $$

Colored histogram pixels indicated the blood reperfusion of ischemic and nonischemic limbs.

### In-vivo capillary density measurement

The capillary density indicates the angiogenesis in the ischemic hindlimb. Generally, mice were sacrificed 14 days after hindlimb ischemic surgery to collect ischemic muscle samples, namely the left gastrocnemius muscle. Then muscle samples were embedded in OCT compound (Sakura, Torrance, CA, USA), frozen by liquid nitrogen, and cut by Leica CM 1950 Cryomicrotome (Carl Zeiss AG, Jena, Germany) at a thickness of 6 μm. Frozen sections were first washed in phosphate-buffered saline (PBS), and then stained with diluted fluorescein-IB4 (Invitrogen, Carlsbad, CA, USA) at room temperature for 1 hour. Samples were then washed in PBS again and sealed with a coverslip. A fluorescent microscope (Olympus, Tokyo, Japan) was used to examine the images.

### EPC isolation and characterization

After ischemic muscle samples were collected, the bones were then separated for EPC isolation. The purified EPCs would be used directly for the following functional determination with no other treatment. The protocol of EPC isolation and culture was described previously [16, 17]. After the muscle sample was collected, the bones were then separated and smashed to collect bone marrow mononuclear cells (BM-MNCs). The bone sample includes hipbones, femurs, and tibiae, as well as shoulder bones, ulnas, vertebra, and sternum. EPCs were further isolated by layering the BM-MNCs on a density gradient (Histopaque 1083; Sigma) followed with centrifugation and were cultured in Endothelial Cell Basal Medium-2 (EBM-2; Lonza, Basel, Switzerland), supplemented with EGM-2 MV SingleQuots (Lonza). After 2 days of culture in a 37 °C, 5% CO_2_ incubator, nonadherent cells were washed off by PBS while adherent cells were further incubated in fresh EBM-2 for 1 week before experiments.

Culture medium was gently washed off by PBS, and then cells were cultured in 5 μg/ml of DiI-Ac-LDL solution (Biomedical Technologies Inc., Stoughton, MA, USA) in EBM-2 for 4 hours at 37 °C, with the dish wrapped in aluminum foil. The cells were then fixed by 4% paraformaldehyde (PFA)/PBS for 20 min at 37 °C. After this, the cells were washed by PBS and DAPI staining solution (Beyotime Biotechnology, Shanghai, China) was added to stain the nuclei. After 10 min of incubation, staining solution were washed off by PBS and the sample was ready to be observed by fluorescent microscope.

### EPC tube formation assay

The tube formation assay was carried out as described previously to determine the in-vitro tube incorporation potential of EPCs [18, 19]. Briefly, human umbilical cord-derived endothelial cells (HUVECs; 1 × 10^4^ cells/well) and DiI-labeled (Invitrogen, Eugene, OR, USA) EPCs (1 × 10^3^ cells/well) were first resuspended using EBM-2 (100 μl), then seeded together on a 96-well plate, with 50 μl Matrigel matrix (BD, Bedford, MA, USA) added to each well beforehand, and incubated for 30 min at 37 °C. A fluorescent microscope was used to take morphology images, to count the number of DiI-labeled cells, and to measure the tube length.

### EPC transwell assay

A modified Boyden chamber assay was used to examine the EPC migration activity [20]: 500 μl of 15% FBS/Dulbecco's modified Eagle's medium (DMEM; Gibco, New York, NY, USA) was added into the lower chamber. EPCs (1 × 10^4^ cells/well) in 200 μl serum-free medium were placed in the upper chamber, with 0.05–0.2% bovine serum albumin (BSA; Amresco, Englewood, CO, USA) in order to maintain the osmotic pressure. The chamber was incubated for 4 hours under 5% CO_2_ at 37 °C. Cells on the lower side of the chamber were fixed with PFA and stained with DAPI. The number of migrated cells was counted using a fluorescent microscope.

### EPC senescence detection

EPC senescence was evaluated based on the quantity of senescence-associated β-galactosidase (SA-β-gal)-positive cells. SA-β-gal was stained using the senescence β-galactosidase staining kit (Beyotime Biotechnology, Shanghai, China), according to the manufacturer’s instructions [21]. The number of SA-β-gal-positive cells was determined by counting blue cells from at least 1000 cells per field.

### Colony-formation assay

Methylcellulose-based medium (MethoCultM3236; Stem Cell Technologies, Vancouver, BC, Canada) was prepared with supplements as follows: VEGF (50 ng/ml; PeproTech, Rocky Hill, NJ, USA), SCF (100 ng/ml; PeproTech), IL-3 (20 ng/ml; PeproTech), EGF (50 ng/ml; PeproTech), bFGF (50 ng/ml; PeproTech), IGF-1 (50 ng/ml; PeproTech), and 30% FBS. EPCs were cultured in this medium for 14 days under 5% CO_2_ at 37 °C. A phase-contrast microscope (Olympus) was used for manual quantitative counting.

### Real-time reverse-transcriptase PCR analysis

Real-time reverse-transcription polymerase chain reaction (RT-PCR) was used to determine the mRNA expression of VEGF and Ang-1 in EPCs and hindlimb muscle. Total RNA was extracted using Trizol reagent (Ambion by Life Technologies, Carlsbad, CA, USA) and then purified according to the manufacturer’s protocol (QIAGEN, Valencia, CA, USA). cDNA was converted from extracted total RNA using the PrimeScript RT reagent kit (TAKARA, Tokyo, Japan). Then, 2 μl of cDNA sample was used as a template for quantitative real-time PCR (RT-qPCR). qPCR was performed using Power Syber Green (Applied Biosystems, Foster City, CA, USA) and the StepOne-Plus real-time PCR system (Applied Biosystems) according to the manufacturer’s guidelines. The endogenous housekeeping gene *GAPDH* was used to normalize the results. The sequences of primers used in qPCR are presented in Table 1. The 2^−ΔΔCT^ method was used to evaluate the relative quantification of changes in the expression of target genes [22].

**Table 1:** Primer sequences for quantitative real-time PCR

Gene name	Forward primer (5′–3′)	Reverse primer (5′–3′)
*mVEGF-A*	AGCACAGCAGATGTGAATGC	AATGCTTTCTCCGCTCTGAA
*mANG-1*	ATCTTGATAACCGCAGCCAC	TGTCGGCACATACCTCTTGT
*GAPDH*	TGTGTCCGTCGTGGATCTGA	ACCACCTTCTTGATGTCATCATACTT

### Statistical analyses

All results are expressed as mean ± SD. The data were analyzed using GraphPad Prism 6.0 software, via Student's unpaired *t* test for the difference between two groups or one-way ANOVA with Bonferroni correction for multiple group comparison. Differences were considered significant at *P* < 0.05.

## Results

### Promoted in-vivo blood flow recovery in curcumin-treated type 1 diabetic mice

**Fig. 1:**
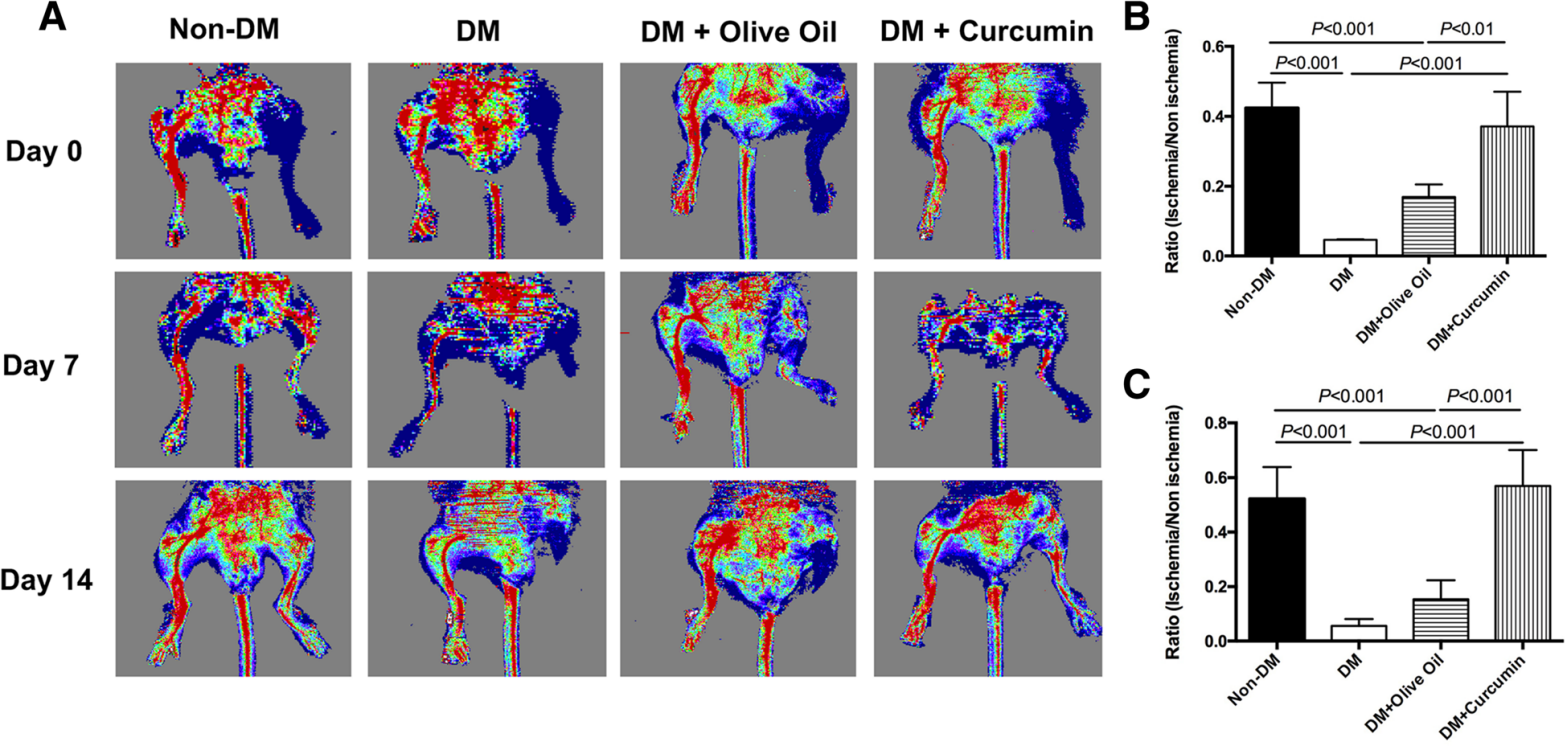
Curcumin promoted blood flow recovery in ischemic hindlimbs of type 1 diabetic mice. **a** LDPI measurement of hindlimb blood flow at day 0, 7, and 14 after ischemic surgery, presented as representative images. Colors represent the perfusion degree: *red*, highest velocity; *green*, intermediate; *blue*, low velocity. **b, c** Quantitative analysis of the blood flow perfusion ratio of ischemic-to-nonischemic hindlimbs after 7 and 14 days respectively (*n* = 5). *non-DM* nondiabetes group, *DM* diabetes with saline-treated group, *DM + olive oil* diabetes with olive oil-treated group, *DM + curcumin* diabetes with curcumin-treated group (Color figure online)

A diabetic mouse ischemia model was constructed to examine the neovascularization ability with curcumin treatment. Blood perfusion was measured by LDPI at day 0, 7, and 14 after the ischemic operation, and the recovery index was presented by the blood flow recovery ratio as mentioned previously [23] (Fig. 1a). In general, blood flow recovery was significantly delayed in the saline-treated and olive oil-treated groups, whereas the blood flow reperfusion in curcumin-treated group presented a dramatic elevation, exhibiting a similar recovery ratio to the nondiabetic group. Specifically, the blood flow recovery ratio in the olive oil-treated group was 16.93 ± 3.60% at day 7 and 15.36 ± 7.04% at day 14 (Fig. 1b, c). In comparison, the ratio in the curcumin group (day 7, 37.17 ± 9.83%; day 14, 56.99 ± 13.02%) had significantly increased (*P* < 0.05), indicating that curcumin could promote blood flow recovery in diabetic hindlimb ischemia.

### Enhanced neovessel density of diabetic ischemic hindlimb

**Fig. 2:**
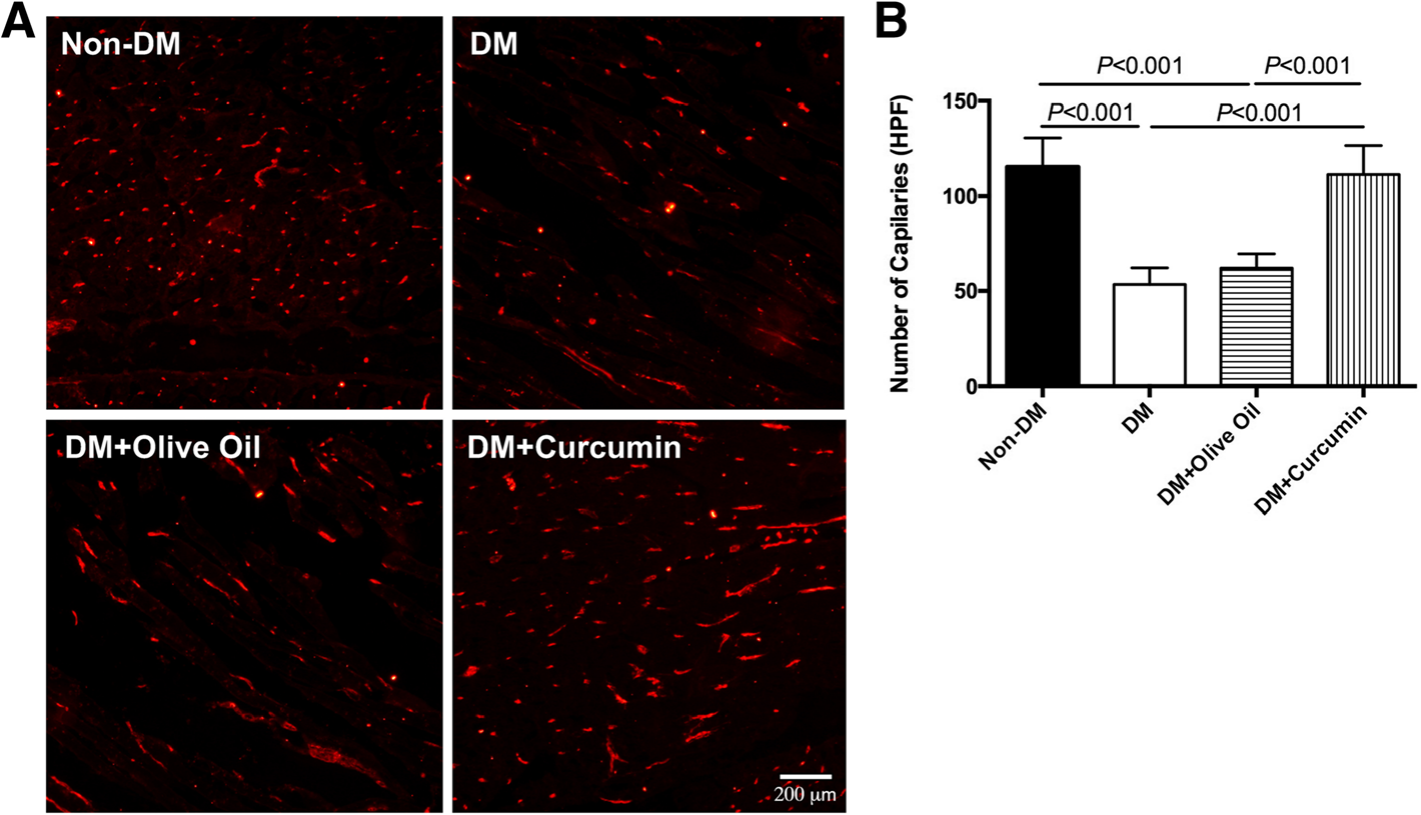
Histological and quantitative analysis of ischemic hindlimb capillary density. **a** IB4-stained cells (*red*) were identified as neogenic capillaries. *Bar*, 200 μm. **b** Numbers of capillaries were counted and presented as mean ± SD (*n* = 4). *HPF* high-power field, *non-DM* nondiabetes group, *DM* diabetes with saline-treated group, *DM + olive oil* diabetes with olive oil-treated group, *DM + curcumin* diabetes with curcumin-treated group (Color figure online)

In addition to LDPI, capillary density was further examined by immunohistochemistry at day 14 postoperatively. The immunofluorescent agent IB4 was applied to endothelial cells. IB4-labeled capillaries were manually counted in high-power fields (HPFs). Overall, EPCs isolated from diabetic mice with saline treatment and olive oil treatment both exhibited insufficient growth of neovessels (Fig. 2; saline group, 53.40 ± 8.79/HPF; olive oil group, 62.00 ± 7.58/HPF). On the contrary, enhanced neovascularization could be observed in the curcumin-treated diabetes group (111.40 ± 15.27/HPF). These data demonstrate that the angiogenesis in diabetic ischemic hindlimb was augmented by curcumin treatment.

### Characterization of EPCs

**Fig. 3:**
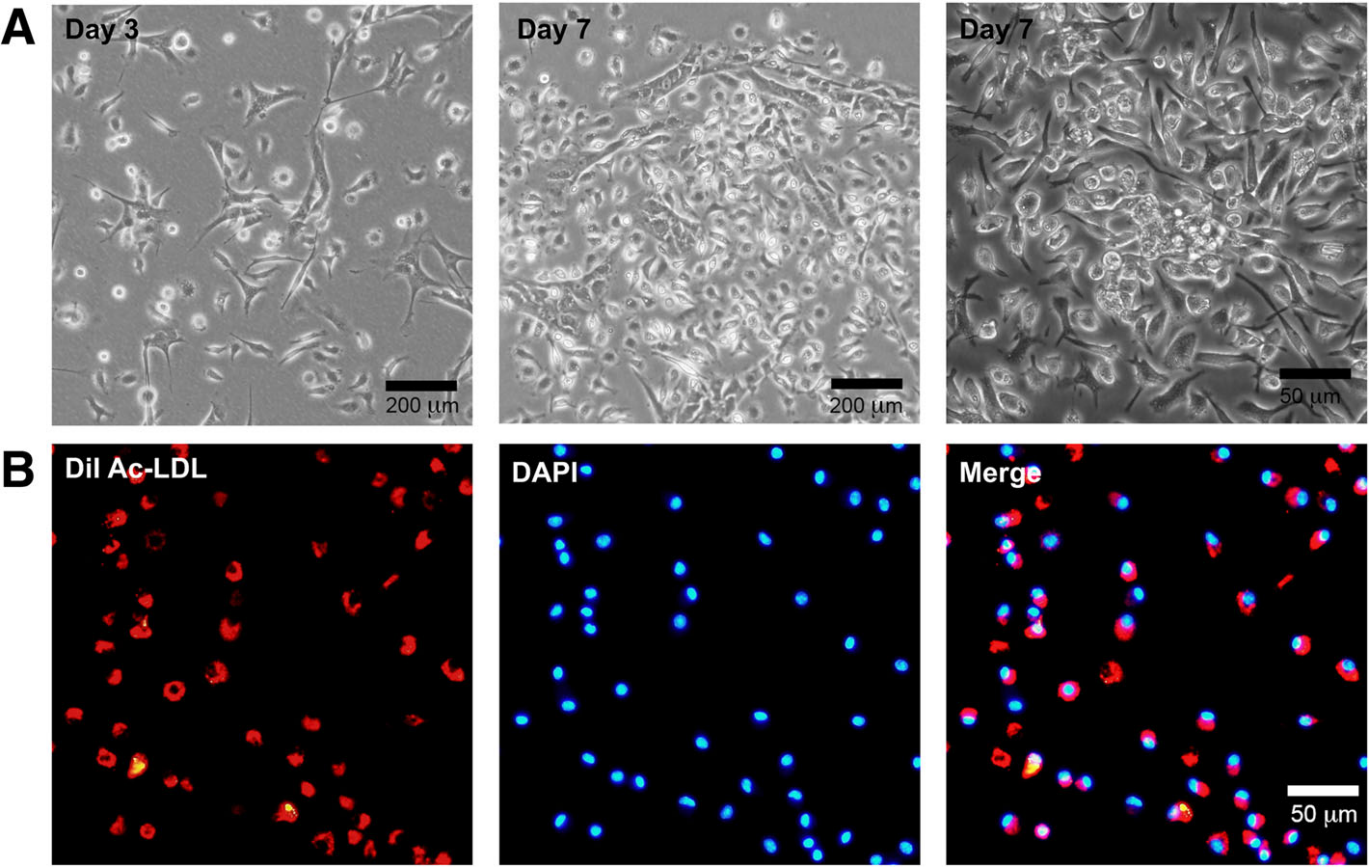
Characterization of BM-derived EPCs. **a** Spindle-shaped EPCs could be observed 3 days after plating BM-MNCs. EPCs showed cobblestone-like morphology at day 7. *Bar*, 200 μm and 50 μm. **b** EPCs uptaking DiI-Ac-LDL could be visualized (*red*). Nucleus (*blue*) was stained by DAPI. *Bar*, 50 μm (Color figure online)

Cells were cultured in medium of 10% FBS/EBM-2MV for 7 days after isolation from mice BM. At day 3, adherent cells changed their morphology into a spindle shape. At day 7, most cells had changed their appearance and formed colonies as reported previously [5, 24] (Fig. 3a). Further investigation was conducted to determine the EPC characteristics by DiI-Ac-LDL uptake assay. Cells positive for uptake DiI-Ac-LDL observed by fluorescent microscope were considered EPCs (Fig. 3b).

### Augmented in-vitro tube incorporation activity of curcumin-treated EPCs isolated from diabetic mice

**Fig. 4:**
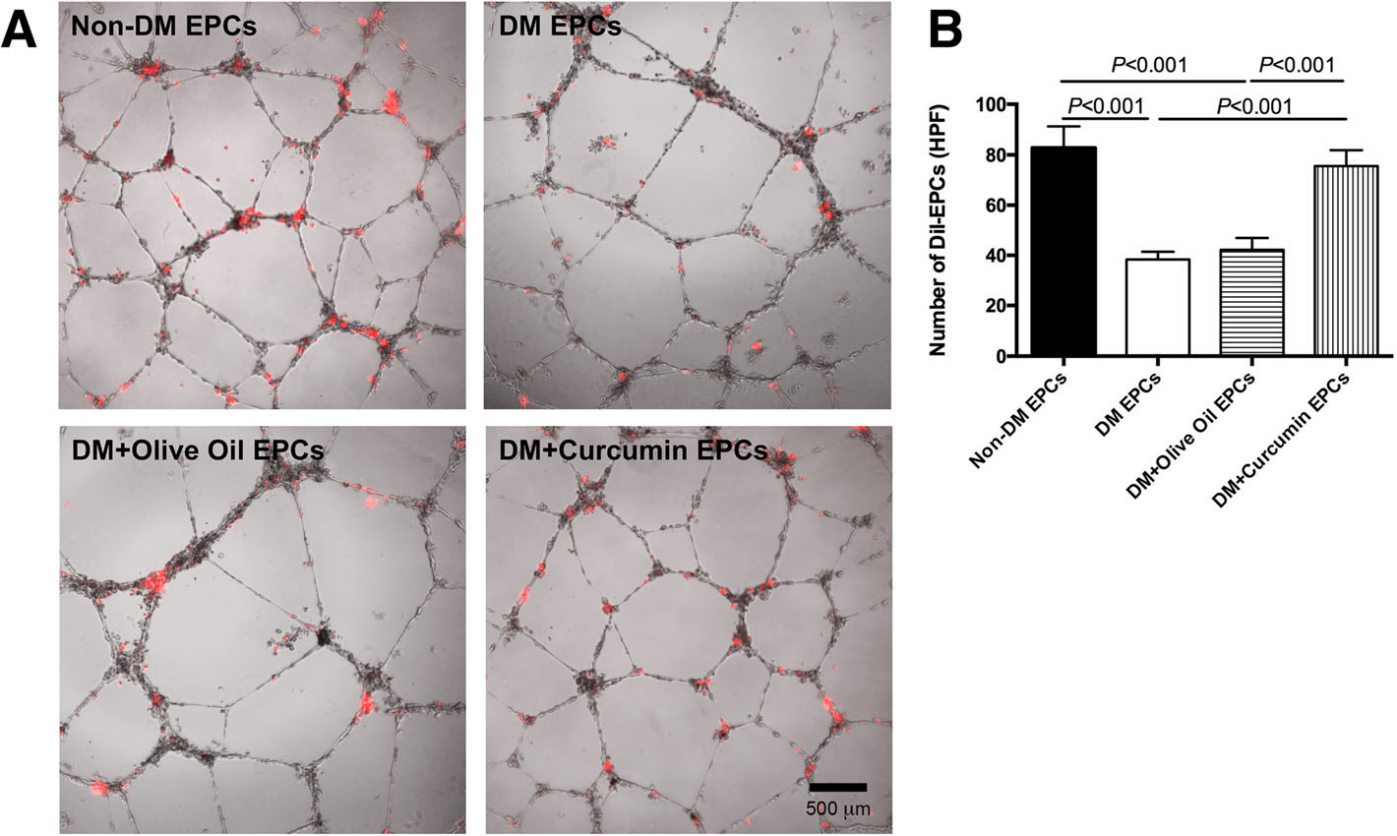
Tube incorporation ability of EPCs isolated from diabetic mice was rescued by curcumin. **a** Morphology images of tube-formed HUVECs incorporated with DiI-positive EPCs isolated from diabetic mice (*red*). *Bar*, 500 μm. **b** Number of DiI-positive incorporated EPCs was counted and presented as mean ± SD (*n* = 4). *EPC* endothelial progenitor cell, *HPF* high-power field, *non-DM* nondiabetes group, *DM* diabetes with saline-treated group, *DM + olive oil* diabetes with olive oil-treated group, *DM + curcumin* diabetes with curcumin-treated group (Color figure online)

The tube formation assay was introduced to study the angiogenesis potential of EPCs according to former reports [19, 25]. EPCs are not capable of forming the tube-like structure independently, but could integrate into the tube-like structure formed by HUVECs. The number of incorporated EPCs was counted manually. As shown in Fig. 4a, EPCs in untreated groups presented impaired tube incorporation capacity compared with that in non-EPCs isolated from diabetic mice. Precisely, 42.20 ± 4.76/HPF incorporated EPCs were observed in the olive oil group, whereas 75.60 ± 6.31/HPF incorporated EPCs were observed in the curcumin-treated group (Fig. 4b). This assay revealed that the curcumin-treated diabetic group showed better EPC incorporation into the new tubes than the olive oil-treated diabetic group.

### Reversed senescence of EPCs isolated from diabetic mice by curcumin application

**Fig. 5:**
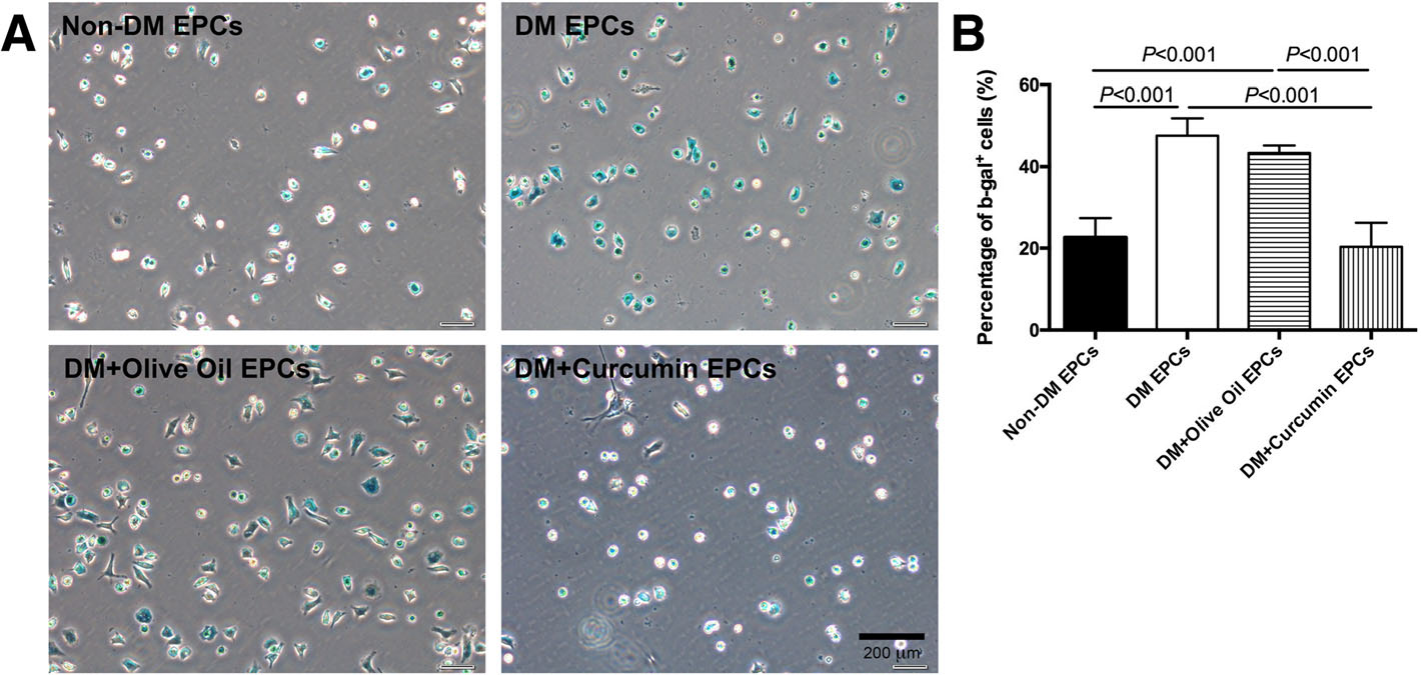
Senescence of EPCs studied using β-galactosidase staining agent. **a** Representative images of SA-β-Gal-positive cells (*blue*). *Bar*, 200 μm. **b** Percentage of β-Gal^+^ cells to total cells was calculated manually and presented as mean ± SD (*n* = 4). *EPC* endothelial progenitor cell, *non-DM* nondiabetes group, *DM* diabetes with saline-treated group, *DM + olive oil* diabetes with olive oil-treated group, *DM + curcumin* diabetes with curcumin-treated group (Color figure online)

EPC senescence is increased under hyperglycemia condition [26]. To test whether curcumin could reverse the accelerated senescence of EPCs, we conducted a SA-β-gal assay. The senescent cells could be visualized in blue (Fig. 5a), and the senescence ratio of EPCs is presented in Fig. 5b (*P* < 0.05; olive oil group, 43.32 ± 1.83% vs curcumin treatment, 20.33 ± 5.90%). These data indicate that curcumin may have the potential to reverse the senescence of EPCs isolated from diabetic mice.

### Promoted migratory ability of curcumin-treated EPCs isolated from diabetic mice

**Fig. 6:**
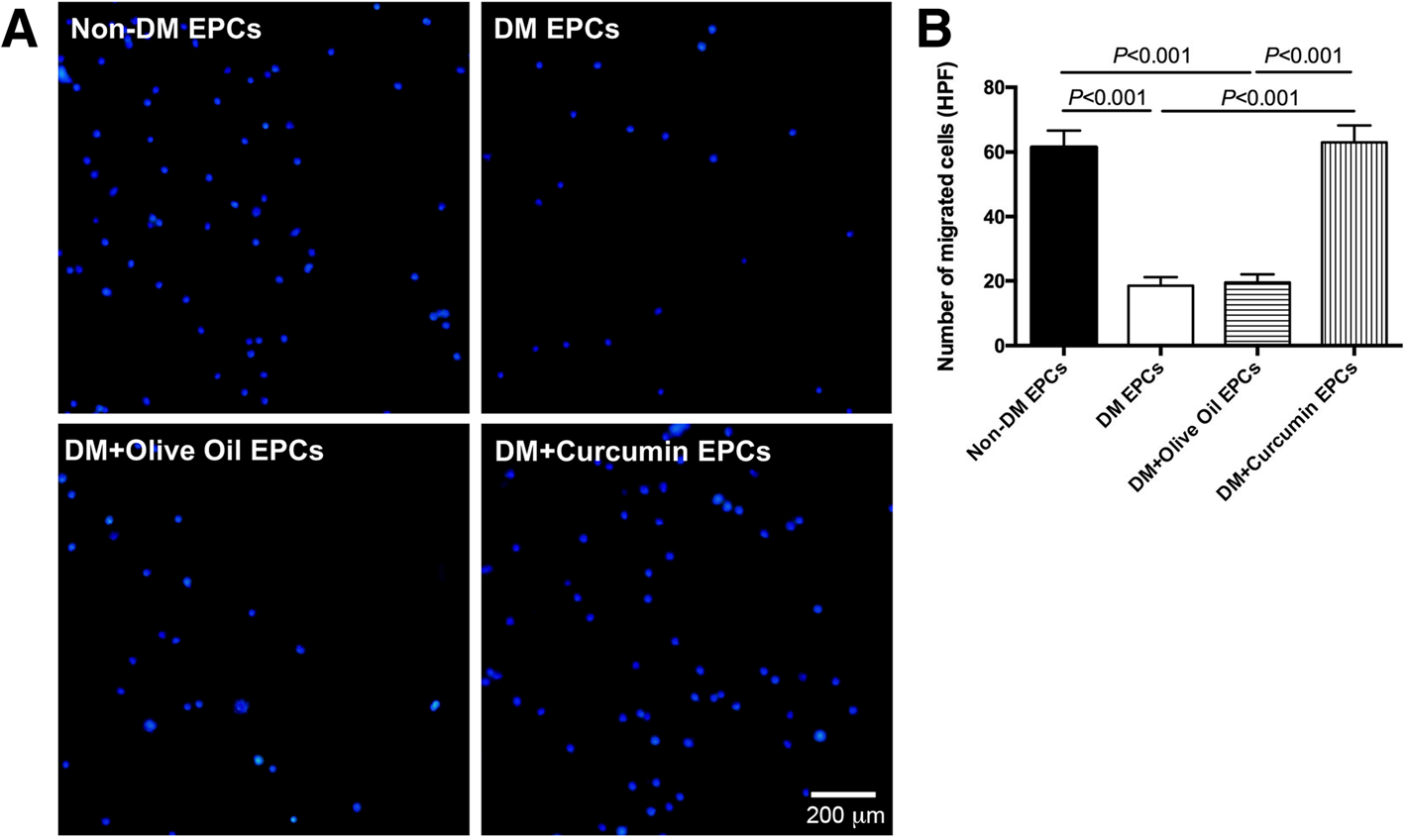
Transwell assay to detect the migratory ability of EPCs isolated from diabetic mice. **a** Representative images show DAPI-stained migrated cells (*blue*) taken by fluorescent microscope. *Bar*, 200 μm. **b** Number of migrated cells was calculated manually and presented as mean ± SD (*n* = 4). *EPC* endothelial progenitor cell, *HPF* high-power field, *non-DM* nondiabetes group, *DM* diabetes with saline-treated group, *DM + olive oil* diabetes with olive oil-treated group, *DM + curcumin* diabetes with curcumin-treated group (Color figure online)

The transwell system was introduced to study the migration ability of EPCs isolated from diabetic mice. Cells were stained by DAPI and observed in HPFs (Fig. 6a). After treatment with curcumin, EPCs isolated from diabetic mice manifested an augment in migration compared with control groups (*P* < 0.05; non-DM, 61.50 ± 5.20/HPF; DM, 18.50 ± 2.65/HPF; DM + olive oil, 19.50 ± 2.52/HPF; DM + curcumin, 63.00 ± 5.29/HPF). This assay indicated that curcumin had the potential to enhance the migration ability of EPCs isolated from diabetic mice.

### Enhanced proliferation ability of EPCs isolated from diabetic mice by curcumin

**Fig. 7:**
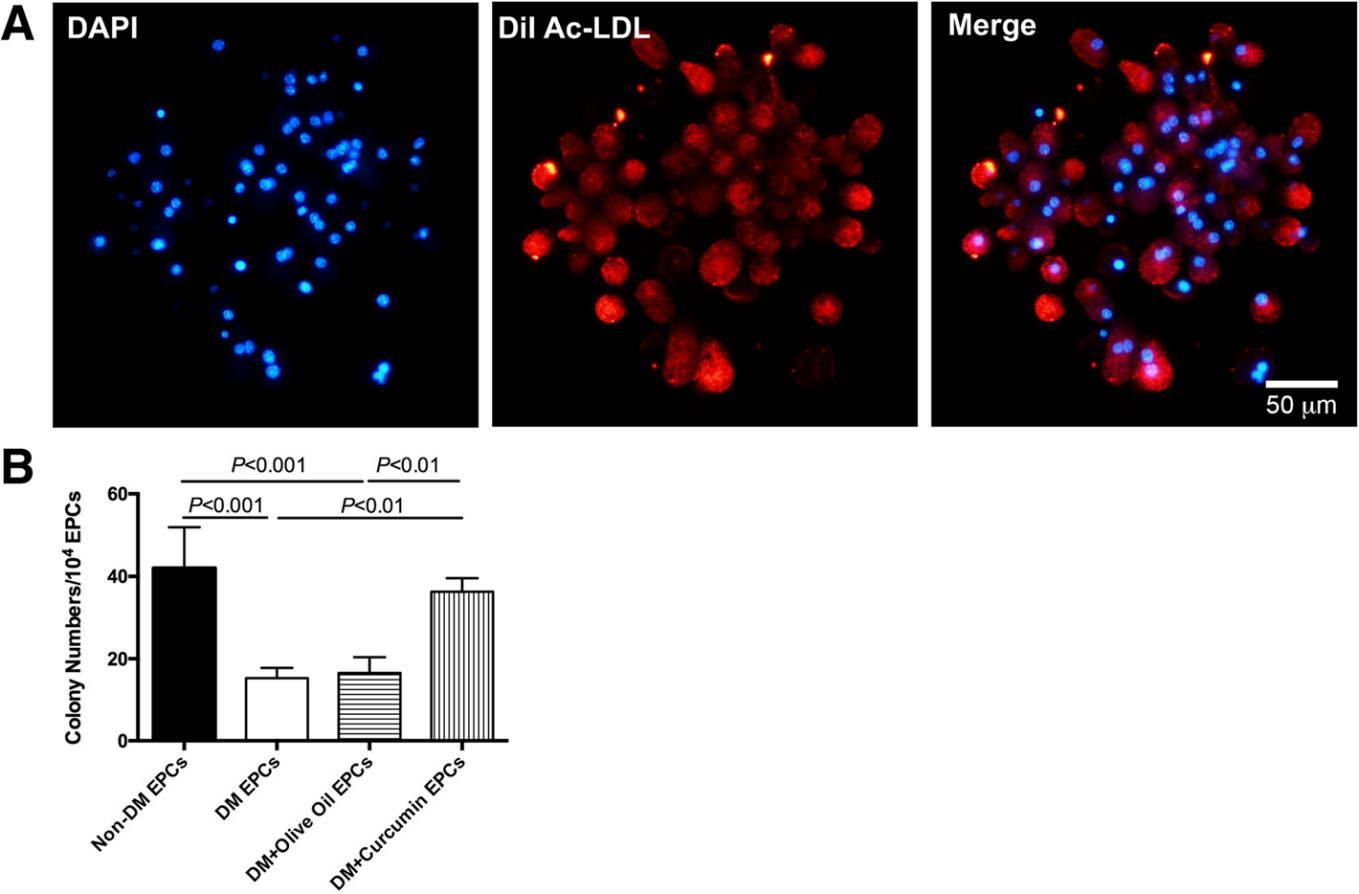
Colony-formation assay showed curcumin could reverse the proliferation ability of EPCs isolated from diabetic mice. **a** Representative staining for DiI-Ac-LDL in EPC colonies. *Bar*, 50 μm. **b** EPC colonies were counted and analyzed in each group. Number of EPCs presented as mean ± SD (*n* = 4). *EPC* endothelial progenitor cell, *non-DM* nondiabetes group, *DM* diabetes with saline-treated group, *DM + olive oil* diabetes with olive oil-treated group, *DM + curcumin* diabetes with curcumin-treated group (Color figure online)

The colony-formation ability of EPCs, representing the proliferation ability, was determined after curcumin application. EPCs in the colonies could uptake DiI-Ac-LDL (Fig. 7a). Curcumin application increased the number of colonies by about twofold compared with untreated groups, without reaching a significant difference compared with the non-DM group (Fig. 7b; non-DM, 42.00 ± 9.90; DM, 16.50 ± 3.87; DM + olive oil, 16.50 ± 3.87; DM + curcumin, 36.25 ± 3.30; *P* < 0.05).

### qPCR validation of VEGF-A and Ang-1 expression

**Fig. 8:**
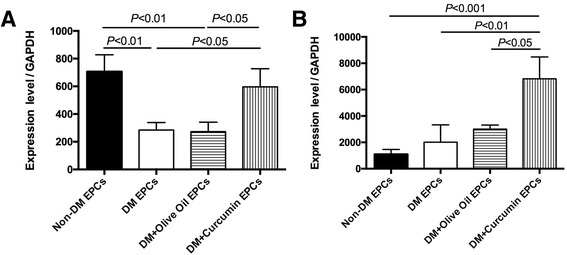
qPCR analysis of neovascularization factors in different EPC groups. mRNA level of (**a**) VEGF-A and (**b**) Ang-1 determined by quantitative real-time RT-PCR. GAPDH used for the normalization of mRNA expression (*n* = 3). *EPC* endothelial progenitor cell, *non-DM* nondiabetes group, *DM* diabetes with saline-treated group, *DM + olive oil* diabetes with olive oil-treated group, *DM + curcumin* diabetes with curcumin-treated group

VEGF-A and Ang-1 are considered the key proangiogenic factors [27]. Thus, expression of VEGF-A (Fig. 8a) and Ang-1 (Fig. 8b) was further analyzed by RT-qPCR. Curcumin could upregulate the expression of VEGF-A and Ang-1 in EPCs isolated from diabetic mice.

## Discussion

In the present study, we demonstrated the curative effects of curcumin on ischemic hindlimbs in diabetic mice via promoting the function of EPCs isolated from diabetic mice. Assays and data exhibited that curcumin application to diabetic ischemia had the potential of: significantly promoting blood flow recovery via enhanced neovascularization; enhancing EPC function, namely angiogenesis, migration, and proliferation ability; reversing the hyperglycemia-induced EPC senescence; and upregulating the angiogenic factors, in terms of VEGF-A and Ang-1, in EPCs.

Kaushik et al. [28] reported spontaneous amputation of lower extremities in diabetes, demonstrating the high risk of necrosis and amputation in DM associated with PAD. In this study, more than half of the ischemic diabetic mice in untreated groups suffered from autoamputation by day 14, while the other half of these mice had different levels of necrosis (Additional file 1: Figure S1), which is in accordance with the above report. In contrast, curcumin application significantly rescued hindlimb ischemia in diabetic mice. The immunofluorescent result indicated that the capillary density was augmented substantially in the curcumin-treated group compared with the untreated DM groups. This result manifested the strong therapeutic effects of curcumin on hindlimb ischemia in diabetes mellitus. We also observed that after the application of curcumin to euglycemic hindlimb ischemic mice, blood flow recovery and the neogenic vessels presented no significant difference from the control group (Additional file 2: Figure S2).

EPCs are involved in the neovascularization under hyperglycemic condition [29, 30]. Based on the in-vivo findings, we proposed that curcumin could take effect via modulating the functions of EPCs in diabetic ischemia. As a follow-up study, we isolated EPCs 14 days after curcumin application and directly detected their functions. We observed that curcumin reversed the impaired angiogenesis, migration, and proliferation abilities and the accelerated senescence of EPCs, and could hypothesize the hindlimb ischemia might be rescued by the augmented functions of EPCs. In order to exclude the potential effects of STZ on murine EPCs, we applied STZ directly to EPCs isolated from healthy mice. There were almost no promotional or inhibitory effects on the proliferation, tube formation, and migration abilities of the cells by STZ (Additional file 3: Figure S3).

One important finding in this study is that curcumin could upregulate the expression of VEGF-A and Ang-1 in EPCs. Kant et al. [11] observed curcumin-induced upregulation of VEGF expression in diabetic wounds. On the contrary, Shehzad et al. and Shan et al. [13, 14] observed that curcumin could downregulate VEGF expression in cancer cells. To explore the reason of these opposite results, Kiran et al. [11] further applied curcumin to endothelial cell culture in serum-free and serum-supplemented conditions, respectively. This study indicates that curcumin presents different effects depending on its microenvironment. Specifically, when cells are exposed to growth factors, curcumin may present an antiangiogenic effect; when cells are in a microenvironment that lacks exogenous stimuli, curcumin may have a proangiogenic effect [27]. Accordingly, we determined the mRNA level of VEGF and Ang-1 in hindlimb muscle collected from diabetic and nondiabetic mice. The mRNA expression level of both VEGF and Ang-1 was lower in the diabetic group than in the nondiabetic group (Additional file 4: Figure S4; VEGF, 722.9 ± 140.6 in non-DM vs 390.7 ± 121.3 in DM; Ang-1, 365.8 ± 135.6 in non-DM vs 106.8 ± 13.9 in DM; *P* < 0.05), indicating a lack of proangiogenic factors in the diabetic microenvironment. To sum up, the microenvironment in hyperglycemia lacks exogenous stimuli factors [2, 3], which explains the phenomenon that in our study curcumin upregulated VEGF expression in EPCs, presenting a proangiogenic effect.

## Conclusion

The results of this study reveal that lavage application of curcumin could ameliorate the decreased blood flow reperfusion in diabetic mice. In addition, EPCs isolated from curcumin-treated diabetic mice showed enhanced ability in migration, angiogenesis, and proliferation, and decreased senescence. Furthermore, VEGF-A and Ang-1 expression was significantly upregulated, which may be induced by curcumin treatment. In conclusion, curcumin manifested its therapeutic effects on the diabetic ischemic mice model, and could possibly be envisioned as a promising therapeutic method in treating diabetic ischemic diseases in human beings.

## Additional files

Additional file 1: Figure S1. is showing gross appearance of mouse hindlimb ischemia model on day 14. Mice were anesthetized and photographs were taken at day 14. (JPG 3289 kb)

Additional file 2: Figure S2. is showing blood flow and the capillary density in ischemic hindlimbs of euglycemic mice treated with or without curcumin. (**A**) LDPI measured hindlimb blood flow at day 7 and 14 after ischemic surgery, presented as representative images. Colors represent the perfusion degree: *red*, highest velocity; *green*, intermediate; *blue*, low velocity. (**B, C**) Quantitative analysis of blood flow perfusion ratio of ischemic-to-nonischemic hindlimb after 7 and 14 days respectively (*n* = 3). LDPI results presented no difference between the group treated with curcumin and the control group. (**D**) IB4-stained cells (*red*) were identified as neogenic capillaries. *Bar*, 200 μm. (**E**) Numbers of capillaries were counted and presented as mean ± SD (*n* = 3). *non-DM*, non-diabetes group; *non-DM + curcumin*, nondiabetes group treated with curcumin. (JPG 2372 kb)

Additional file 3: Figure S3. is showing tube incorporation ability (**A**), migratory ability (**B**), and colony formation ability (**C**) of EPCs isolated from euglycemic mice treated with or without STZ. (**A**) Number of DiI-positive incorporated EPCs was counted and presented as mean ± SD (*n* = 4). Tube incorporation ability of EPCs treated with STZ presented no difference with the control group. (**B**) Number of migrated cells was calculated manually and presented as mean ± SD (*n* = 4). EPCs treated with STZ presented similar migratory ability to the control group. (**C**) EPC colonies counted and analyzed in each group. Number of EPCs colonies presented as mean ± SD (*n* = 4). *STZ* streptozotocin. (JPG 1294 kb)

Additional file 4: Figure S4. is showing qPCR analysis of VEGF and Ang-1 in diabetic and nondiabetic mice hindlimbs. Total mRNA level of (**A**) VEGF-A and (**B**) Ang-1 determined by quantitative real-time RT-PCR. GAPDH was used for the normalization of mRNA expression (*n* = 3). (JPG 1841 kb)

## Abbreviations

Ang-1: Angioprotein-1
BM: Bone marrow
BM-MNC: Bone marrow mononuclear cell
DM: Diabetes mellitus
EPC: Endothelial progenitor cell
HPF: High-power field
LDPI: Laser Doppler perfusion imaging
PAD: Peripheral arterial disease
SD: Standard deviation
VEGF: Vascular endothelial growth factor
