# Supplementary figures and images for: Curcumin induces therapeutic angiogenesis in a diabetic mouse hindlimb ischemia model via modulating the function of endothelial progenitor cells
Source: Stem Cell Res Ther. 2017 Aug 3;8:182. doi: 10.1186/s13287-017-0636-9 (PMC5543575; doi:10.1186/s13287-017-0636-9)

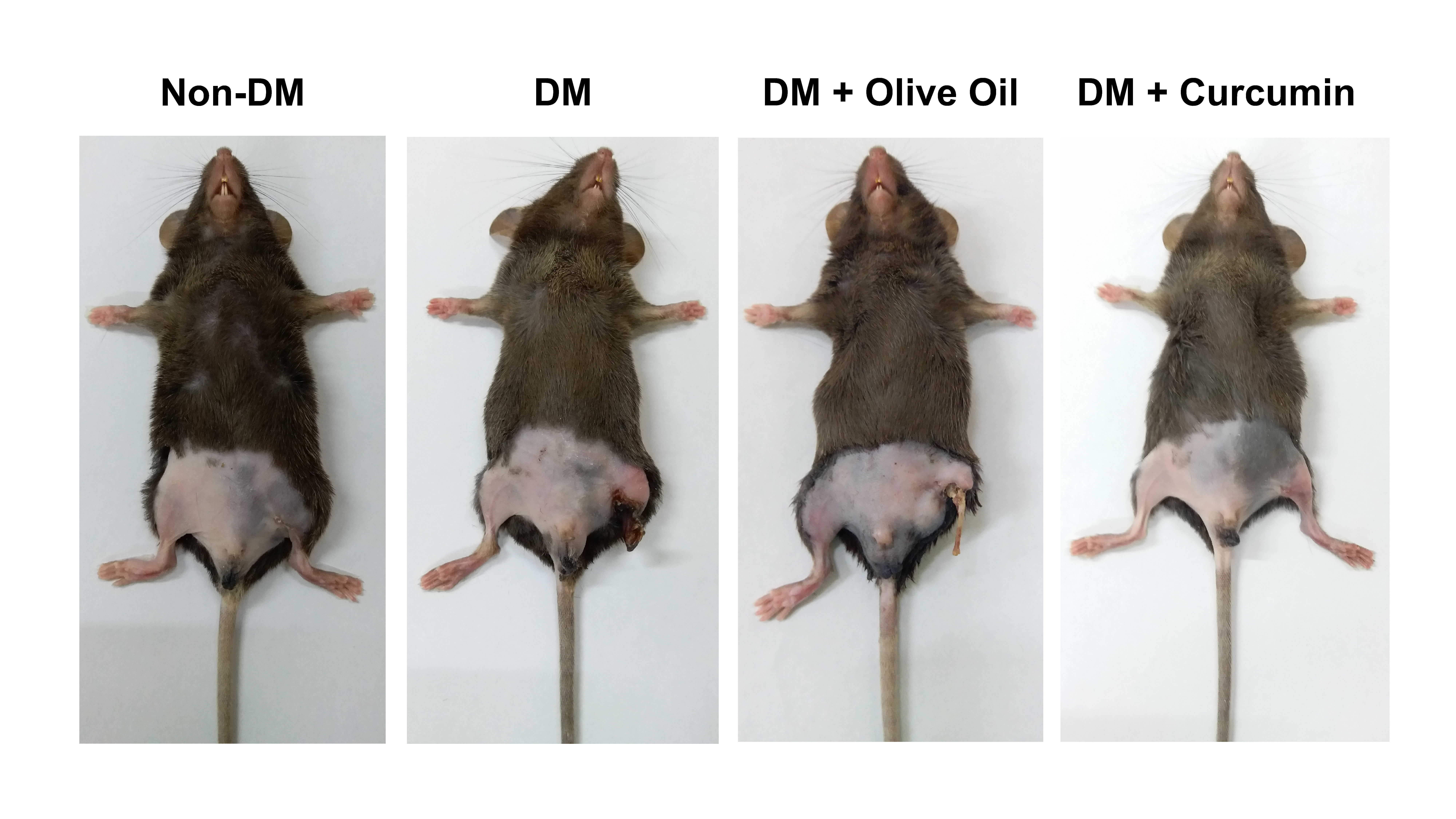

Supplement: Supplementary file 1 — is showing gross appearance of mouse hindlimb ischemia model on day 14. Mice were anesthetized and photographs were taken at day 14. (JPG 3289 kb) [file 13287_2017_636_MOESM1_ESM.jpg]

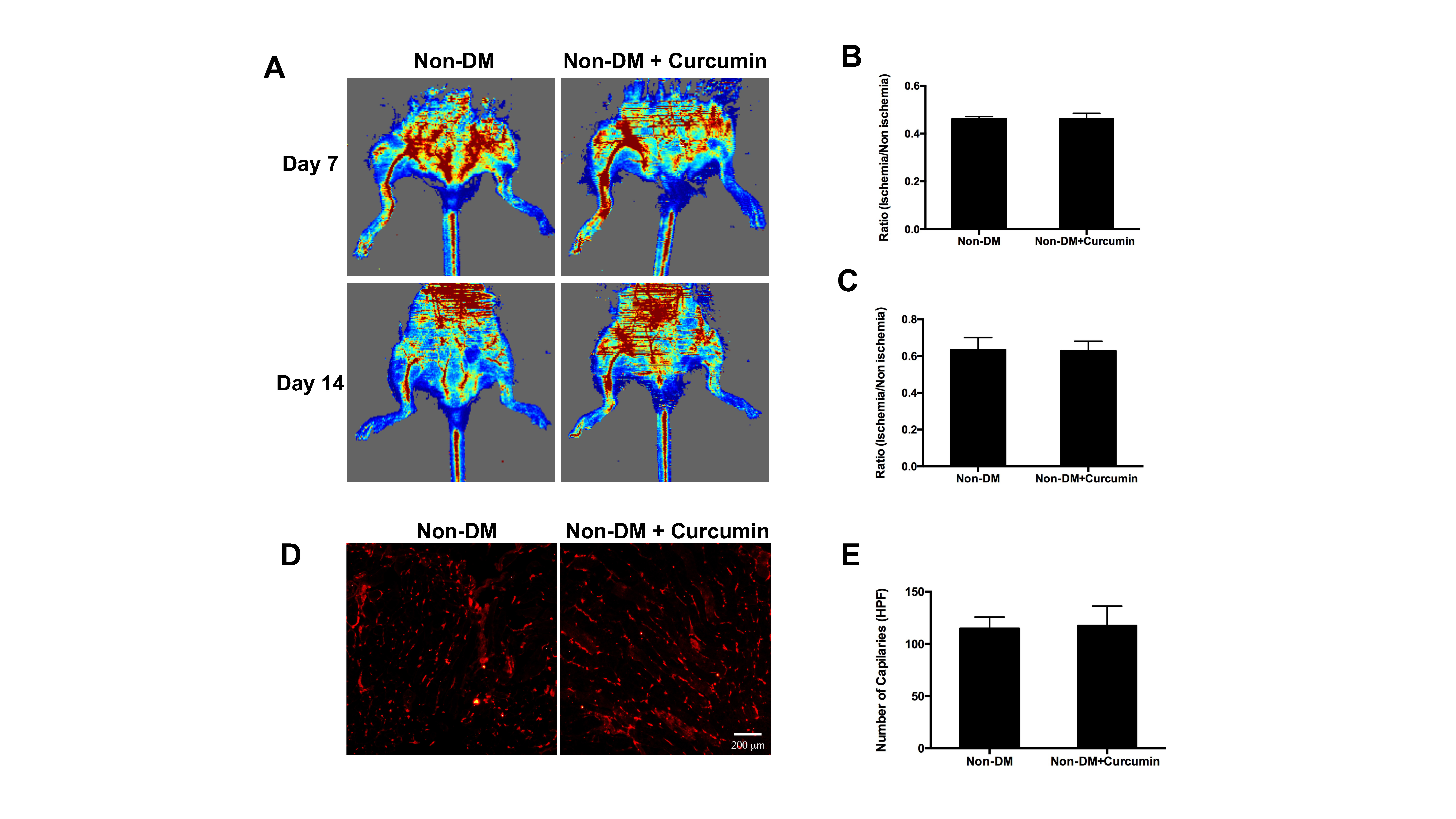

Supplement: Supplementary file 2 — is showing blood flow and the capillary density in ischemic hindlimbs of euglycemic mice treated with or without curcumin. (A) LDPI measured hindlimb blood flow at day 7 and 14 after ischemic surgery, presented as representative images. Colors represent the perfusion degree: red, highest velocity; green, intermediate; blue, low velocity. (B, C) Quantitative analysis of blood flow perfusion ratio of ischemic-to-nonischemic hindlimb after 7 and 14 days respectively (n = 3). LDPI results presented no difference between the group treated with curcumin and the control group. (D) IB4-stained cells (red) were identified as neogenic capillaries. Bar, 200 μm. (E) Numbers of capillaries were counted and presented as mean ± SD (n = 3). non-DM, non-diabetes group; non-DM + curcumin, nondiabetes group treated with curcumin. (JPG 2372 kb) [file 13287_2017_636_MOESM2_ESM.jpg]

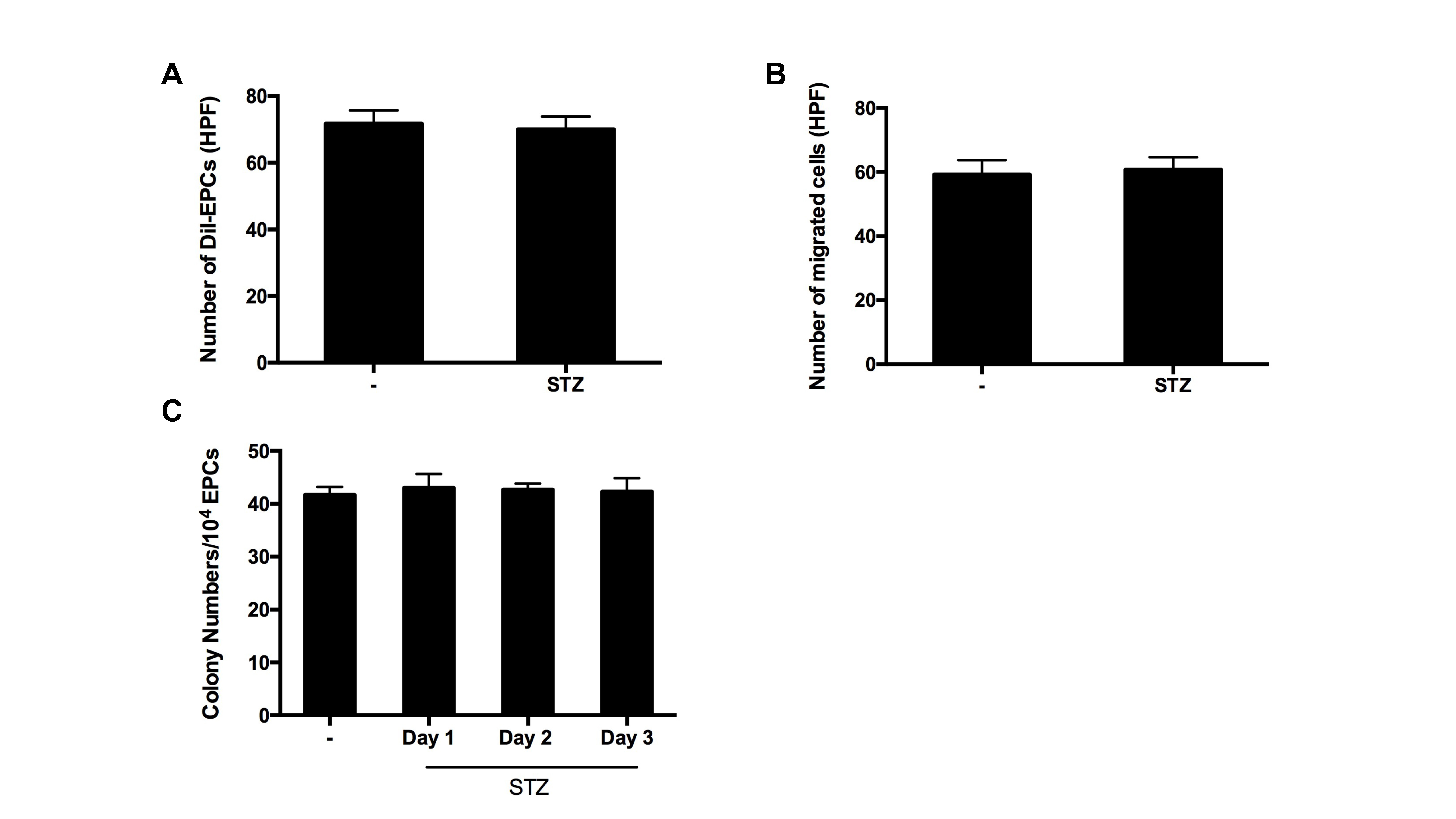

Supplement: Supplementary file 3 — is showing tube incorporation ability (A), migratory ability (B), and colony formation ability (C) of EPCs isolated from euglycemic mice treated with or without STZ. (A) Number of DiI-positive incorporated EPCs was counted and presented as mean ± SD (n = 4). Tube incorporation ability of EPCs treated with STZ presented no difference with the control group. (B) Number of migrated cells was calculated manually and presented as mean ± SD (n = 4). EPCs treated with STZ presented similar migratory ability to the control group. (C) EPC colonies counted and analyzed in each group. Number of EPCs colonies presented as mean ± SD (n = 4). STZ streptozotocin. (JPG 1294 kb) [file 13287_2017_636_MOESM3_ESM.jpg]

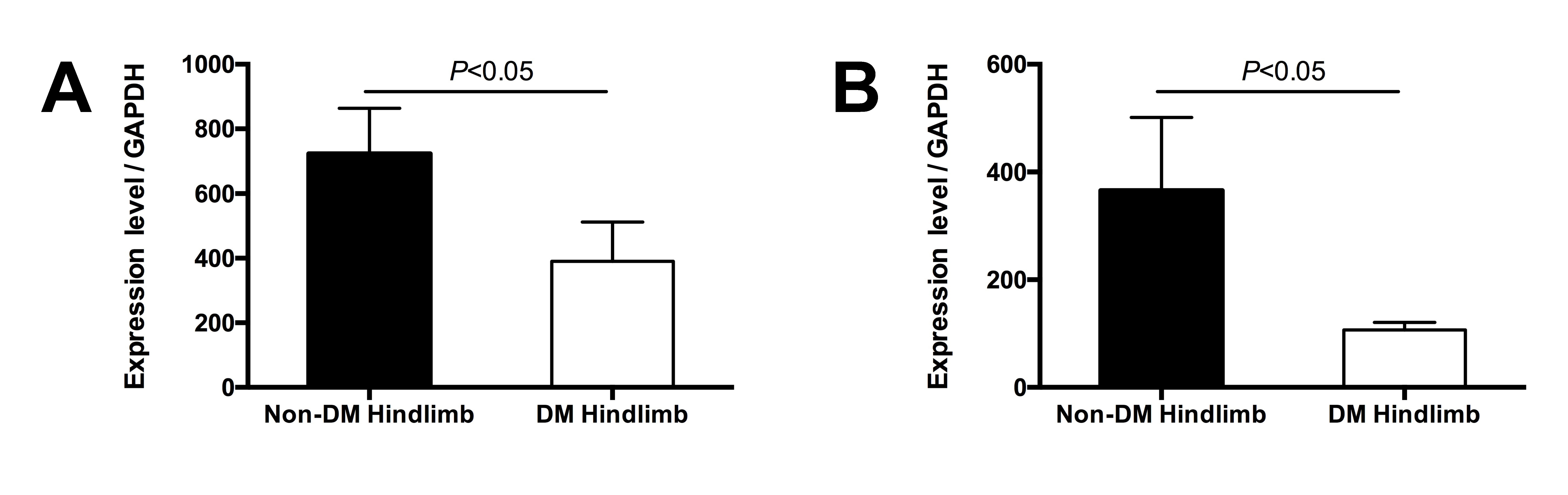

Supplement: Supplementary file 4 — is showing qPCR analysis of VEGF and Ang-1 in diabetic and nondiabetic mice hindlimbs. Total mRNA level of (A) VEGF-A and (B) Ang-1 determined by quantitative real-time RT-PCR. GAPDH was used for the normalization of mRNA expression (n = 3). (JPG 1841 kb) [file 13287_2017_636_MOESM4_ESM.jpg]
